# Analysis of QoS Requirements for e-Health Services and Mapping to Evolved Packet System QoS Classes

**DOI:** 10.1155/2010/628086

**Published:** 2010-10-07

**Authors:** Lea Skorin-Kapov, Maja Matijasevic

**Affiliations:** Faculty of Electrical Engineering and Computing, University of Zagreb, Unska 3, HR-10000 Zagreb, Croatia

## Abstract

E-Health services comprise a broad range of healthcare services delivered by using information and communication technology. In order to support existing as well as emerging e-Health services over converged next generation network (NGN) architectures, there is a need for network QoS control mechanisms that meet the often stringent requirements of such services. In this paper, we evaluate the QoS support for e-Health services in the context of the Evolved Packet System (EPS), specified by the Third Generation Partnership Project (3GPP) as a multi-access all-IP NGN. We classify heterogeneous e-Health services based on context and network QoS requirements and propose a mapping to existing 3GPP QoS Class Identifiers (QCIs) that serve as a basis for the class-based QoS concept of the EPS. The proposed mapping aims to provide network operators with guidelines for meeting heterogeneous e-Health service requirements. As an example, we present the QoS requirements for a prototype e-Health service supporting tele-consultation between a patient and a doctor and illustrate the use of the proposed mapping to QCIs in standardized QoS control procedures.

## 1. Introduction

With recent trends and technology advancement in the development of converged broadband next generation networks (NGNs) and advanced multimedia services, the potential has increased for delivering various e-Health services to end users “anywhere, anytime”. The term e-Health has been used to refer to the use of information and communication technology (ICT) in delivering healthcare services [1]. A wide variety of e-Health services exist, including health information networks, electronic health record (EHR), telemedicine services, wearable and portable systems which communicate, health portals, and many other ICT-based tools assisting disease prevention, diagnosis, treatment, health monitoring, and lifestyle management. A related term is m-Health, referring to “mobile computing, medical sensor, and communications technologies for health care” [2]. M-Health services refer to e-Health services in mobile environments, characterized by limited resource availability and changing network conditions [3].

**Figure 1:**
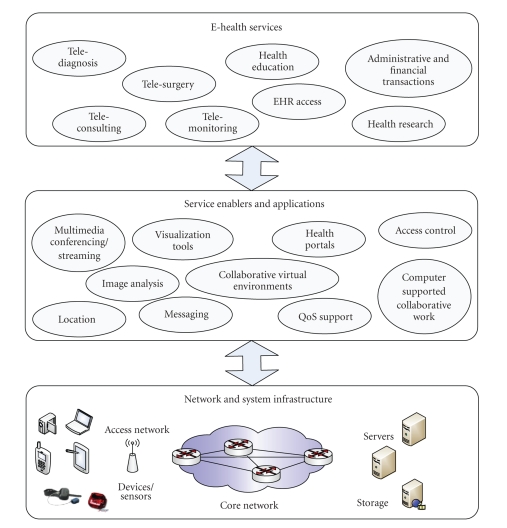
Layered service environment for e-Health services.

In general, a wide variety of services may be built on top of tools and applications that provide the necessary communications and computer-aided support (e.g., multimedia conferencing/streaming enablers, image analysis and visualization tools, immersive and collaborative virtual environments, etc.), as shown in Figure 1.

Converged NGNs are being designed to deliver different types of traffic across heterogeneous end-user environments. In order to meet the requirements of e-Health service traffic delivered over networks in conjunction with other commercial traffic (e.g., voice calls, streaming multimedia, and Internet traffic), QoS mechanisms such as class-based traffic prioritization are necessary. The wide variety of e-Health services impose different Quality of Service (QoS) requirements on underlying networks. One aspect is delay tolerance, with service requirements ranging from strict real-time and delay-intolerant data transmission (e.g., tele-consultation services involving transmission of patient physiological parameters in emergency situations) to delay-tolerant services (e.g., access to a patient's EHR; home tele-monitoring). Another aspect is application data sensitivity to loss, with conversational voice-based applications often tolerating a certain packet loss, while data transmission (e.g., transfer of medical images) being highly loss intolerant. A significant amount of related work deals with performance requirements of e-Health services and evaluated network capabilities in meeting those requirements. In [4], the authors categorize the importance of various QoS parameters for different fields of e-Health. Prioritization and resource allocation schemes for various types of telemedicine traffic delivered over wireless networks has been addressed in [5, 6]. Further studies have more specifically focused on evaluating support for the delivery of emergency telemedicine services over high speed 3G networks [3, 7–10] and other wireless networks [11, 12], with evaluation results showing generally reliable performance. Apart from emergency scenarios, 3G networks have been evaluated in the support of various tele-consultation services involving the delivery of high-definition images [13], such as the delivery of ultrasound still and streaming images in robotic tele-ultrasonography systems [14]. Projects such as MobiHealth [15], HealthService24 [16], and MyHeart [17] have focused on developing systems for continuous tele-monitoring of patient vital signals and their transmission to healthcare institutes using 2.5/3G networks. (It should also be noted that within the European Seventh Framework Programme there are many more projects focusing on e-Health services [18], but they do not specifically consider their provisioning and QoS in 3G networks.) While this list is by no means exhaustive, it demonstrates the emerging needs which the NGN aims to answer.

Limited research, however, has focused on evaluating support for e-Health services in the context of the latest NGN standards. In order to support multimedia service delivery over a multiaccess converged all-IP core network, the Third Generation Partnership Project (3GPP) has finalized the Release 8 specifications of the Evolved Packet System (EPS), thus representing a milestone in the development of standards for the mobile broadband industry [19]. For a detailed description of EPS, an interested reader is referred to [20]. The EPS represents an evolution of the 3G UMTS characterized by higher data rates, lower latency, and a packet-optimized system aimed to deal with the rapid growth in IP traffic. A key element of the EPS is specification of a class-based QoS control concept offering service and subscriber differentiation [21]. The packet forwarding treatment received by a given session data flow is based on an assigned QoS Class Identifier (QCI) that serves as a standardized reference to node-specific treatment (e.g., scheduling weights, admission thresholds, queue management thresholds, etc.). The 3GPP specifications include nine QCIs with corresponding standardized characteristics in terms of bearer type (guaranteed versus nonguaranteed bit rate), priority, packet delay, and packet-error-loss rate.

In the context of delivering e-Health services, a key issue for the EPS QoS control architecture will be the accurate mapping of service requirements to QCIs. We emphasize that our focus in this paper is not on determining the actual network requirements of e-Health services, as a significant amount of related work deals with this issue. Rather, we aim to contribute to the ongoing research by proposing a mapping of requirements to 3GPP QCIs, based on a classification of heterogeneous e-Health service context and network QoS requirements. The proposed mapping aims to provide network operators with valuable guidelines for enabling service prioritization and making necessary network resource authorization decisions. The paper is organized as follows. In Section 2, we discuss the various requirements of e-Health services and propose a service classification.  Section 3 gives a short overview of the 3GPP QoS control architecture. A mapping of e-Health service requirements to standardized QCIs is given in Section 4.  Section 5 presents an example involving a tele-consultation service between a patient and a doctor used to illustrate EPS QoS control procedures and use of the proposed mapping to QCIs.

## 2. QoS Requirements for e-Health Services

### 2.1. E-Health Service Classification

Among the numerous classifications of e-Health services that may be found in literature, services are often broken down based on specific objectives into the following [13]: tele-diagnosis, tele-consultation, tele-monitoring, tele-management, tele-education, and value-added services. *Tele-diagnosis* services have been described as generally characterized by asynchronous point-to-point communication (e.g., specialists at a remote site review transmitted patient data and return a diagnosis report), while *tele-consultation* has been described as generally based on synchronous viewing and manipulation of medical multimedia data. *Tele-monitoring* in most cases refers to transmission of a patient's vital bio-signals and other related data, as in the case of home care telemedicine services [22]. Such services are often targeted at treating patients with chronic diseases or for posthospital home care, and may involve multiparametric monitoring including patient vital signs (e.g., electrocardiogram (ECG), blood pressure, saturation of peripheral oxygen (SpO2), glucose level, etc.), physical sensors (monitoring patient activity), and environmental sensors (e.g., air temperature, humidity, and air pressure). The European Commission funded MobiHealth project has focused on mobile tele-monitoring.Tele-monitoring may also involve an expert interacting with a remote examination site using audio/visual communication. For the purposes of this paper, we use the term *tele-education* as referring to any health-related education performed at a distance and in non-emergency situations. In [13], Perakis and Koutsouris use the term *tele-management* to refer to a combination of advanced tele-monitoring and tele-consultation services, such as those involving computer assisted medical interventions and automatic surgical tools (tele-surgery).

A classification of e-Health services based on QoS requirements has been proposed in Vouyioukas et al. [7]. The authors state that applications may generally be classified as real-time applications and near real-time applications. We note, however, that in certain cases the instances of the same generic type of service (e.g., tele-diagnosis) may have very different QoS requirements depending on actual context in which the service is invoked. For example, in an emergency situation, a remote specialist diagnosis may require near real-time transmission of medical data, while in a different, non-emergency situation, the patient medical data is transferred (with tolerance for delay) to a remote location to be analyzed by specialists. Another example of a service with strict QoS requirements and involving patient critical data transmission is tele-surgery. Hence, determining service context in terms of emergency or patient critical versus non-emergency and noncritical service delivery is crucial in providing input for traffic scheduling mechanisms. Context awareness with respect to QoS has also been addressed for m-Health services [23], where the authors use contextual information (information about the user environment) to adapt the service.  Table 1 illustrates the classification proposed in [7], extended by the notion of sensitivity to context, whereby context refers to the emergency nature of the service. All application types for which use in an emergency or patient critical context may be envisioned are marked as being sensitive to context. We build upon this idea later, in the proposed mapping of QoS requirements to QCIs.

### 2.2. QoS Requirements for Typical e-Health Services

Typical e-Health applications may involve multimedia conferencing, transmission of patient physiological parameters, transfer of high resolution medical images, transmission of clinical/administrative data, and access to EHRs. Such applications generate traffic with very diverse network requirements, differing in required bandwidth, real-time versus non-real-time interactivity, and tolerance for packet loss. Often times, an e-Health service will involve the simultaneous transmission of multiple media flows, such as for example a mobile emergency system including audio/video, medical images, and ECG signals. In this section, we present an overview of related work that has focused on specifying the requirements of such services.

#### 2.2.1. Multimedia Conferencing

Multimedia conferencing applications are often a key part of e-Health services, as they may be used for various communication scenarios including patient-doctor, doctor-doctor (e.g., hospital specialists and general practitioners), and patient-patient scenarios (e.g., virtual support groups). Furthermore, they may involve preorchestrated, as well as live conferencing [24]. In general, voice and video transmission applications tolerate some packet loss as a tradeoff for achieving low-delay and real-time interactivity. The Third Generation Partnership Project (3GPP) specifies the requirements for conversational audio/video applications in UMTS networks as being highly delay and jitter sensitive, with one-way end-to-end (E2E) delay bounds being 150–400 ms [25]. With regards to loss, acceptable frame erasure rates (FERs) are specified as <3% (voice) and <1% (video). Furthermore, the International Telecommunications Union (ITU) specifies objective values for IP packet transfer performance in IP networks, with bounds of 100–400 ms for E2E delay and 1 × 10^−3^ packet loss ratio for real-time conversational services [26]. The ITU also specifies the model for end-user QoS categories with respect to tolerance to information loss and delay tolerance, and provides indicative performance targets for audio and video applications as well as for data applications [27]. The 3GPP has specified the quality of experience and related metrics of the end-to-end multimedia service performance in 3G networks [28].

The particular requirements for multimedia conferencing used in telemedicine depend on service context. For example, a service involving audio/video conferencing between a patient and a doctor for a routine checkup may be considered “less critical” with regards to QoS guarantees (i.e., may tolerate increased degradation and delays of 150–400 ms) as compared to an audio/video conferencing service employed in an emergency accident situation where visual communication with a remote specialist doctor is imperative (i.e., “hard” real-time interactivity with one-way delay 0–150 ms).

In [7], the authors note that it is important to distinguish between the requirements for: (a) real-time video transmission, (b) offline video transmission, (c) medical video and audio for diagnostic applications, and (d) nondiagnostic video and audio. Real-time video transmission for diagnostic applications is stated as being the most demanding. Real-time diagnostic audio applications include the transmission of stethoscope audio, or the transmission of the audio stream that accompanies the diagnostic video.

#### 2.2.2. Still and Streaming Medical Images

The transmission of high definition still images is often a part of a tele-consultation service. Examples of images include: dermatological images, X-Rays, Magnetic Resonance Images (MRIs), ultrasound images, and computed tomography (CT) [13]. With regards to bandwidth, there are no specific requirements other than the fact that low bandwidth leads to longer transmission times. An overview of image sizes and data rates corresponding to typical devices is given in Table 2 (taken from [7]). In general, an important issue in the transfer of medical data is reliable data delivery, with packet losses having potentially disastrous consequences in terms of patient diagnosis.

#### 2.2.3. Tele-Robotic Systems

Tele-robotic systems, such as those used for tele-surgery and tele-ultrasonography, may involve the transmission of both still and streaming images. QoS requirements are generally very strict in terms of delay and loss intolerance, with invasive robotic services (tele-surgery) being patient critical and thus having more stringent requirements than noninvasive robotic services (e.g., tele-ultrasonography).

In the case of robotic tele-surgery, a key requirement is a minimal delay time from when a surgeon's hand movement is initiated, the remote manipulator actually moves, and images are shown on the surgeon's monitor [29]. Studies have shown that the limit of the acceptable time delay in terms of a surgeon's perception of safety was roughly 330 ms [30]. Mechanisms for compensating delay include slowing surgeon hand movement and a remote surgeon performing tasks that require less precision, while a local surgeon performs precision-dependent tasks. Furthermore, it has been noted that two-way video conferencing among members of the healthcare team greatly enhances robotic tele-surgery [29]. With regards to reliability and error rate, relatively low data rates for transmission of robotic control data (<20 kbps) allow for error-protection coding and the possibility for transmitting equipment to send commands more than once to the receiving end [31].

The QoS requirements of a robotic tele-ultrasonography system have been conducted in the scope of the end-to-end mobile tele-echography using an ultralight robot (OTELO) project [14]. The project developed a fully portable tele-operated robot allowing a specialist sonographer to perform a real-time robotized tele-echography (ultrasonography) to remote patients. Three types of critical data are transmitted over the OTELO system: (1) robotic control data, (2) ultrasound still images, and (3) medical ultrasound streaming data, with controlled ultrasound medical streams being the most demanding in terms of data rate (in that case QCIF format and H.263 codec have been used). Focusing on a UMTS network, the authors point out that for the exchange of medical image sequences with real-time requirements, a mapping to the UMTS *Conversational* QoS class would be necessary. A test carried out on the OTELO system showed reliable functioning of the system with a minimum packet loss of less than 0.5 percent. Furthermore, performance evaluation of the ultrasound streaming images showed that round trip delays (along the expert-patient-expert path) of up to 300 ms were within acceptable boundaries of maintaining high/quality real-time interaction of the system.

#### 2.2.4. Transmission of Patient Vital Signs

The amount and frequency of information related to monitored patient vital signs that needs to be transmitted depends on patient needs. While for some patients it may be sufficient to transmit vital signs every few minutes, other patients (e.g., those considered high-risk) may require transmission every few seconds. In [32], the authors discuss the requirements of tele-monitoring systems for cardiac patients which consist of wearable and light-weight wireless biomedical sensors (for measuring 3 lead ECG, SpO2, heartbeat, and blood pressure). Sensors communicate with a signal processing module which further transmits physiological measurements (based on patient-specific thresholds, timing and frequency as specified by a healthcare provider) via various network interfaces to, for example, hospital servers, emergency stations, local physician clinic, and so forth. Transmission requirements are mapped to the following categories based on the severity of the patient's health condition (as specified by a health provider):

- Class 0: highest priority requiring real-time monitoring (patients in emergency situations, or, with severe medical conditions);
- Class 1: requiring near real-time monitoring within a few hours;

(iii) Class 2: requiring periodic monitoring such as twice daily;

(iv) Class 3: requiring monitoring from time to time.

The MobiHealth project [15] developed a system for the continuous monitoring of patient vital signals (using body area networks) and their transmission to healthcare institutes using GPRS and UMTS. Trials were conducted involving home care, high-risk patient monitoring, and emergency services, with the goal being to evaluate whether 2.5/3G communications technologies can support the requirements of such systems. Different trials were conducted to cover a range of bandwidth requirements (low: less than 12 kbps, medium: 12–24 kbps, and high: greater than 24 kbps), and to address both non-real-time (e.g., periodic transmission of ECG) and real-time transmission requirements (e.g., alarms, transmission of vital signs in emergency situations) [33]. At the time the trials were run (2003), the identified network barriers included restricted available data bandwidth for uplinks (in tele-monitoring systems, high data rates generally originate at user side, not server), delay variation, delays in transmission (ranging from approximately 100 ms for packet sizes of 174 bytes, to 1200 ms for packet sizes of 8122 bytes), and handover (sometimes resulting in connection loss).

#### 2.2.5. Findings for Emergency e-Health Services

One of the most important application areas for telemedicine that relies on broadband services has been recognized as tele-consultation and tele-diagnosis in emergency accident situations, where paramedics attending to accidents do not have the necessary expertise to handle such situations [8, 13]. This results in the need for real-time transmission of accident victim's physiological parameters (e.g., ECG leads, oxygen saturation, and blood pressure) from an accident site or ambulance vehicle to a hospital/medical center. Furthermore, the transmission of still images and video streaming of the victim to specialized doctors may be of critical importance for the doctor to obtain a thorough clinical image of the patient prior to arrival at the emergency room. Hence, such services generally involve the simultaneous transmission of multiple media types.

The joint transmission of voice, real-time video, ECG signals, and medical scans from an ambulance to a hospital in a realistic cellular multiuser simulation environment based on UMTS is further considered in [8], with corresponding QoS requirements summarized in Table 3. Streaming video traffic is modeled based on measurements of H.263 encoded video. A three-lead ECG signal is sampled at 250 Hz and quantized with 12 bits per sample. While voice and video packets are considered error tolerant, ECG and file transmission require data integrity. In their simulations, the authors set a maximum allowed delay of 400 ms for voice and video traffic and a maximum delay of 300 ms for ECG traffic. The results have shown that UMTS was capable of meeting the set requirements.

Similar research conducted in [9] provides experimental evaluation of a mobile tele-trauma system capable of simultaneously transmitting video, medical images, and ECG signals in real 3G network conditions. Various stream parameters have been tested, including different sampling rates, frame rates, resolutions, and so forth. Images and video were compressed using JPEG and M-JPEG, respectively. The authors note that trauma specialists have suggested that a resolution of 320 × 240 (TV resolution) is enough for trauma cases, while a lower resolution of 160 × 120 may be used in extreme bandwidth conditions. With regards to requirements and stream priorities, the authors conclude the following:

- video requirements: loss tolerant, delay intolerant, and low priority;

(ii) image requirements: loss intolerant, delay tolerant, and medium priority;

(iii) ECG requirements: loss and error intolerant, high priority.

The same traffic priority order as used in [9] has been used by the authors in [6], who present new scheduling ideas for the integration of telemedicine traffic with other traffic types in a high capacity cellular network, focusing on handling urgent telemedicine traffic transmission with full priority, while satisfying the QoS requirements of regular traffic as well. The four types of telemedicine traffic that were considered by the authors in their simulations: ECG, X-ray files, medical images, and video. Their corresponding characteristics are as follows [6]:

- ECG data: sampled at 360 Hz with 11 bits/sample precision. A strict upper bound of 1 channel frame (12 ms) is set for the transmission delay of an ECG packet.
- X-ray file: typical file size is 200 Kbytes. The upper bound for the transmission delay of an X-ray file is set to 1 minute.
- Medical images: files sizes range between 15 and 20 Kbytes/image. The upper bound for the transmission delay of an image is set to 5 seconds.
- Video: H.263 is reported as the most widely used video-encoding scheme for telemedicine video. Traces were used with mean bit rates of 91 Kbps, peak rates of 500 Kbps and standard deviation of 32.7 Kbps. Due to the need for very high-quality telemedicine video, the maximum allowed video packet dropping probability was set to 0.01%.

The performance obtained by using simulation, with telemedicine traffic set to 10% of total channel capacity, showed delay and loss values far below the upper bounds set for the particular data type.

In related work [7], the authors studied the capabilities of a High-Speed Packet Access (HSPA) 3G network in meeting the QoS requirements of emergency situations involving the joint transmission of voice, real-time video, medical data such as ECG and other vital signals, heart sound, and file transfer. Their results showed that in the case of congestion, congestion control and service prioritization may be used based on modifications in the operation of the HSDPA scheduler (critical e-Health services are treated favorably in comparison with all other kinds of calls). By prioritizing emergency e-Health services, the authors show that delay is constrained within acceptable values ranging from 150 ms to 240 ms in the downlink (for VoIP and video, resp.), and approximately 200 ms, 500 ms, and 800 ms in the uplink (for VoIP, medical data, and video, resp.).

In [3], the authors study the QoS requirements of a patient tele-monitoring system for emergency vehicles using 3G UMTS access and propose adaptive QoS decision mechanisms in light of varying network resources. They identify different types of services (audio, video, biomedical signals, transmission of high resolution images, transmission of administrative data, and remote EHR access) which can be combined in different ways based on resource availability to deliver an optimal tele-monitoring service. Combined service QoS (corresponding to simultaneous transmission of different service types in real time) is evaluated against the following thresholds (determined based on ITU standards and additional referenced work): E2E delay threshold for audio as 150 ms and video 250 ms, and packet loss rate as less than 12% audio and less than 10% video. In their previous work [34], the authors have developed an automated tool to model e-Health service requirements, and optimize application design regarding available network resources.

In [5], the author presents a resource allocation model for wireless healthcare information systems which maps e-Health applications to three different service classes based on the emergency nature and degree of interactivity (real time versus nonreal time). The classes include: (1) highest priority class incorporating life-threatening situations, characterized by very low blocking probability; (2) medium priority class representing real-time e-Health applications which are not life-critical, with the possibility of QoS degradation in order to meet the high priority class requirements; and (3) low-priority class representing non-real-time applications whose QoS requirements are met when given resources are not required by the other two classes. Simulation results serve to illustrate the benefits of assigning different priority levels to traffic based on the specific medical application requirements.

#### 2.2.6. Access to Electronic Health Records

Existing and emerging hospital and primary health care information systems are based on the use of electronic health records (EHR). An EHR is designed to contain all possible health relevant data of a person. Over the past years, European governments have identified the EHR as the basis for nation-wide exchange and seamless integration of patient data. Access to and management of EHRs may occur in both emergency and non-emergency situations. Network delay is dependent on the amount of information that is being transmitted. However, a key requirement is reliable transmission with zero packet loss.

#### 2.2.7. Research and Education

A wide variety of applications support health related education, such as distance learning for health professionals located in rural and remote areas [35]. Examples of applications include interactive collaborative tools and tele-conferencing, streaming audio/video, virtual classrooms, and interactive surgical simulations. Such applications are generally not considered to be as time-critical as those involving patient care, and may tolerate low delay, data loss, and unavailability. However, highly interactive surgical simulations would greatly suffer from long delays [4].

Furthermore, biomedical research may involve the transmission of high-resolution images from remote databases. In the case of remote instrument manipulation for research purposes, low-delay requirements may result from the need to position samples or adjust instrument settings [4].

#### 2.2.8. Summary

A summary of findings related to the QoS requirements for e-Health services is given in Table 4. We group together services based on delivery requirements (real time or nonreal time) and transmission type (two-way conversational communication, unidirectional streaming, interactive request-response, and background data retrieval). For certain services, delay requirements are indicated as “not available” since no specific requirements have been found. For example, in the case of image transfer, delay will depend on image size and available bandwidth. It is clear that for emergency services, such transfer should be completed within a few seconds.

A general conclusion based on referenced work is that QoS mechanisms in NGNs are necessary in order to be able to guarantee that the requirements of e-Health services will be met, in particular for emergency and patient critical services. In the following sections, we describe the QoS control architecture specified by 3GPP and map e-Health services to standardized QoS classes.

## 3. QoS Control in the 3GPP EPS

 In order to provide support for IP multimedia services in converged NGNs, the 3GPP has specified the EPS, comprised of both an Evolved Packet Core (formerly known as Service Architecture Evolution (SAE)), together with an evolved radio access network (E-UTRA and E-UTRAN, commonly associated with the Long Term Evolution (LTE) work item) [19]. The EPS also supports non-3GPP access, wireline (e.g., xDSL, cable), as well as fixed and mobile wireless (e.g., WLAN, WiMAX).

**Figure 2:**
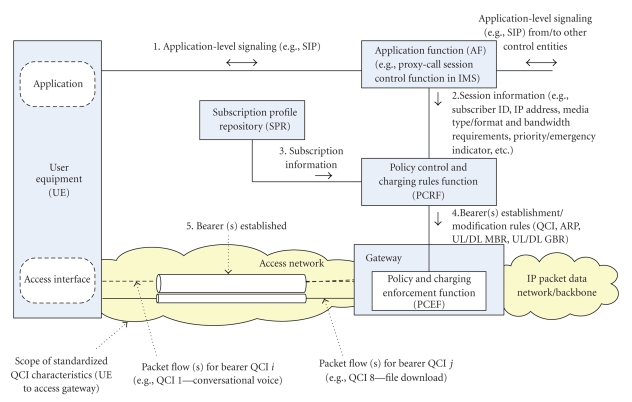
QoS control in 3GPP EPS policy and charging control architecture.

The EPS specifies class-based QoS provisioning, allowing operators to differentiate the treatment received by different subscribers and services. Functional network entities and interfaces responsible for providing service-aware QoS control have been specified as a part of the overall 3GPP Policy and Charging Control (PCC) architecture [36], illustrated in Figure 2, and briefly summarized next. In general, the PCC architecture extends the architecture of an IP-CAN (IP Connectivity Access Network), where the Policy and Charging Enforcement Function (PCEF) is a functional entity in the gateway node implementing the IP access to a packet data network (PDN). An Application Function (AF) located along the application-level signaling path interacts with end user applications, situated in the User Equipment (UE), and extracts session information from signaling flows. An example of an AF is the Proxy-Call Session Control Function (P-CSCF) in the IP Multimedia Subsystem (IMS). The IMS has been specified by the 3GPP (and further adopted by other standardization bodies) as a multimedia session control subsystem comprised of core network elements for the provision of multimedia services [37]. In IMS, session QoS negotiation procedures are based on an end-to-end message exchange using the Session Initiation Protocol (SIP) [38] in combination with the Session Description Protocol (SDP) [39]. An enhancement involving negotiable QoS based on advanced QoS parameter matching and optimization functionality to be included along the signaling path in the IMS has been proposed in [40].

Once the UE is switched on, a default bearer is established, based on subscribed QoS profile. Additional bearers are subsequently established and modified as needed. As shown in Figure 2, the session information is extracted by the AF (1), and is further passed to a Policy Control and Charging Rules Function (PCRF) (2), which is the policy engine of the PCC architecture. The PCRF makes session-level policy decisions to determine whether the user session can have access to requested services and, if yes, under what constraints. Decision-making is based on the session information received from the AF (2), combined with the subscription information/policies for a given user received from a Subscription Profile Repository (3), and the information about access network technology (received from the access network; not shown in the figure). The PCRF then provides session-level policy decisions to the PCEF (4) in the access gateway, where the policy decisions are enforced and used to establish a new bearer or modify an existing bearer (5). Detailed QoS signaling procedures are specified for establishing and modifying bearers [41].

In the scope of the EPS, a particular “bearer” is used to uniquely identify packet data flows belonging to a logical IP transmission path that receive a common QoS treatment between the terminal and the gateway at the edge of the access network. Hence, the bearer is the basic enabler for providing differential treatment for traffic with differing QoS requirements. According to standards, it shall be possible to apply QoS control on a per service data flow basis. The two types of bearers that have been defined are guaranteed bit-rate (GBR) and non guaranteed bit-rate (non-GBR). In the case of a GBR bearer establishment, network resources are reserved in the network (e.g., by an admission control function in a radio base station), and as long as traffic along such a bearer conforms to the reserved GBR, it is assumed that no congestion-related packet loss will occur. On the other hand, services delivered over a non-GBR bearer may experience congestion-related packet loss. Furthermore, a non-GBR bearer may be established for a longer period of time as it does not block transmission resources. A Maximum Bit Rate (MBR), defined as the upper limit for allowed bit rate on a given bearer, may be defined only for GBR bearers. An aggregate MBR (AMBR) values may also be defined for a group of non-GBR bearers (for uplink and downlink separately), thus enabling operators to limit the total amount of bit rate consumed by a single subscriber. GBR bearers are outside the scope of AMBRs. Figure 2 shows an example how different bearers correspond to different packet flows for the given IP address of the end user terminal (one bearer may be established per combination of IP address and QoS class).

Each established bearer is assigned one and only one QoS Class Identifier (QCI). A QCI is defined as a scalar value that represents *a standardized reference* to specific packet forwarding behavior to be provided to a service data flow on the path between a user equipment and access gateway. (The parameters that control the forwarding behavior are preconfigured by the operator owning the node.) The goal of standardizing QCI characteristics is to ensure that applications and services mapped to that particular QCI receive the same minimum level of QoS across multivendor networks, in multioperator environment, and in case of roaming. The 3GPP specifications include nine QCIs with corresponding standardized characteristics in terms of bearer type (also referred to as “resource type”), priority, packet delay budget, and packet-error-loss rate (given in Table 5). A primary difference between QCI 1–4 and QCI 5–9 is the bearer type (GBR versus non-GBR). The specified packet delay budget defines an upper bound for the time that a packet may be delayed between a user equipment and the access gateway, with actual packet delays—in particular for GBR traffic-expected to be typically lower as long as the end user has sufficient radio channel quality. The packet error loss rate defines an upper bound for a rate of noncongestion related packet losses.

Each QCI is further associated with a priority level (from 1 to 9, with priority level 1 being the highest). Priority levels are used to differentiate between service data flow aggregates of the same UE and also to differentiate between flow aggregates from different UEs (i.e., a scheduler shall meet the packet delay budget requirements of flows on priority level *N* in preference to meeting the packet delay budget of flows on priority level *N* + 1).

While a QCI specifies user-plane treatment for associated bearers, the QoS parameter Allocation and Retention Priority (ARP) (also signaled by the PCRF to the access gateway) specifies control plane-treatment for bearers, that is, it may be used to decide whether a bearer establishment or modification request should be accepted or rejected due to resource limitations. The ARP parameter contains information about the priority level, the pre-emption capability and the preemption vulnerability of a resource request. The priority level defines the relative importance of a bearer request. The range of the ARP priority level is 1 to 15, with 1 as the highest level of priority. Values reserved for intraoperator use (priority levels 1–8) may be used to prioritize IMS emergency calls [42]. The pre-emption capability information defines whether a service data flow can get resources that were already assigned to another service data flow with a lower priority level. The pre-emption vulnerability information defines whether a service data flow can lose the resources assigned to it in order to admit a service data flow with a higher priority level. Both values are flags which can be set to either “yes” or “no”. In situations when the system is overloaded, or, when resources must be freed up for other purposes (e.g., an incoming emergency call), bearers associated with a low ARP are released. For example, for video telephony, the operator may map video to a bearer with a lower ARP and voice to a bearer with a higher ARP, and thus have the option to drop only the video bearer if needed, while keeping the voice bearer unaffected. In normal circumstances, ARP has no impact on packet forwarding treatment for successfully established bearers.

Each EPS bearer QoS profile comprises the parameters QCI and ARP; and for GBR bearers also GBR and MBR. For aggregate (set of) EPS non-GBR bearers, AMBR values may be defined. A mapping of authorized IP QoS parameters received from the PCRF to authorized UMTS QoS parameters is performed by the translation/mapping function in the packet gateway. The rules for this mapping with regards to the QCI parameter are specified in [41] and summarized in Table 6. For the purposes of this paper, we assume that the EPS as such can provide the performance as specified and we use these values as a basis for our mapping.

## 4. Mapping of e-Health Service Requirements to Standardized QCIs

In the context of delivering e-Health services over an NGN architecture based on the EPS, a key issue for operators will be the accurate mapping of service requirements at session establishment/modification time to standardized QCIs. A particular service may comprise multiple media types and traffic flows that may need to be mapped to different QCIs. (An example of such a situation is shown in an illustrative example later in this paper.) Using as a basis the analysis of referenced work which has addressed the QoS requirements of heterogeneous e-Health services (summarized in Table 4), we explored the idea of mapping the previously defined types of e-Health services to QCIs. While for some types of e-Health services this mapping turned out to be rather straightforward, the question of context, as well as “relative importance” between flows belonging to different services within the same QCI, proved to be more difficult, as will be explained in more detail shortly. In order to address the requirements of e-Health in different contexts, we find it necessary to break down existing classifications as proposed in [7, 13] by considering service delivery requirements (real time or nonreal time) and transmission type (two-way conversational communication, unidirectional streaming, interactive request-response, and background data retrieval).

Furthermore, certain types of e-Health services mentioned in Table 4 are broken down into multiple e-Health classes based on service prioritization (emergency versus non-emergency). In the case of emergency situations (e.g., medical data transmission from ambulance or accident site to a hospital), data streams should be treated as parts of an emergency session, implying specific call-handling mechanisms and guaranteed QoS support [42]. Emergency service support available in current networks generally refers to emergency calls established in the circuit switched domain, such as 112 or 911 voice calls. With regards to the packet switched domain, emergency IP flows need to be identified by the P-CSCF and signaled to the PCRF (using an emergency indicator) to allow the PCRF to prioritize emergency service data flows over non-emergency service data flows within the access network. In addition to assigning a QCI value, an ARP value may be specified that is reserved for intra-operator use of emergency calls. In general (not only for emergency services) during congestion times the ARP parameter may be used to assign greater priority to bearer establishment/modification for e-Health services, as compared to other typical commercial services (e.g., non-health related calls, networked games, IPTV, etc.).

The proposed mapping is summarized in Table 7, and explained next.

We assume tele-consultation services to be based on synchronous two-way communication between involved parties based on conversational audio and/or video. Such services generally impose large bandwidth requirements and are delay-intolerant and loss-tolerant. We therefore map *real-time conversational voice-based tele-consultation *and *real-time conversational video-based tele-consultation* to QCIs 1 and 2 respectively, with the resource type corresponding to GBR.

As the timely and reliable delivery of e-Health services may in certain cases be considered of critical importance (i.e., a patient's well being or life is endangered), it is imperative that the top priority be assigned to corresponding traffic flows. We argue that in order to support e-Health services, particular classes need to be further broken down with regards to the assigned priority level. We therefore propose for QCI 2 to be broken down with respect to priority level into priority level 2 (for higher priority conversational video-based services) and priority level 4 (standard 3GPP priority level for QCI 2).

Since QCI 3 specifies a GBR and very strict delay bounds (50 ms), we map hard real-time interactive services to this class. We identify the requirements of *invasive real-time robotic services* (e.g., tele-surgery involving the transfer of robotic control data and streaming medical images) as corresponding to QCI 3 characteristics, but distinguish such services from *non-invasive real-time robotic services* (e.g., ultrasound examination) in terms of priority. We therefore break down QCI 3 in two classes corresponding to priority levels 1 and 3. (The priority level 1 in this context should be understood as the “first priority application data”, not the “overall first priority” which is reserved for IMS signaling. In Table 7, this is denoted as priority level 1*.) A potential problem with mapping real-time interactive services to QCI 3 is that the specified packet error loss rate (PELR) for QCI 3 is 10^−3^, which is considered too high for critical services such as tele-surgery. We have previously mentioned that error protection mechanisms may be deployed. It is interesting to note that in the case where more strict loss requirements must be met, the only mapping that “fits” in terms of both delay and loss is that to QCI 5. Considering that QCI 5 is normally used for IMS signaling (signaling indication “yes”), the operator would need to implement a special policy and resource dimensioning to secure the network resources necessary to accommodate both the signaling and emergency application traffic. (The application traffic can be distinguished from the signaling traffic by setting the Signaling Indication to “no”).

Tele-monitoring services generally refer to services involving the transmission of a patient's vital biosignals and other related data. We distinguish between three types of tele-monitoring services based on delay, loss, and bit rate (GBR versus non-GBR) requirements. We map *emergency real-time tele-monitoring* services to QCI 4 and priority level 1 (1*, as noted above), assuming applications involving the streaming of patient vital signs in emergency situations and with very strict loss bounds. A delay of 300 ms may be considered acceptable. *Non-emergency real-time tele-monitoring* services are mapped to QCI 4 and priority level 5 and refer to tele-monitoring services that are not of an emergency nature, but that involve a doctor viewing the patient data in real time. Finally, *non-real-time tele-monitoring services* that are based on patient data which do not involve real-time viewing being delivered to a remote location are mapped to QCI 8 because they are delay tolerant and may be assigned a non-GBR bearer. An example of such a service is patient care, for example, for people with special needs, involving the continuous monitoring of patients at their point of need (e.g., home, work, and on the move).

We map *real-time EHR data access*, *real-time tele-diagnosis*, and *real-time messaging services* to the equivalent of QCI 5 (with same arguments regarding IMS signaling as above) due to sensitivity to loss, as well as a generally interactive (request-response pattern of the end user, rather than conversational or one-way streaming) and high-priority nature of such services. Such services do not require for bearer resources to be blocked for an extended period of time (as is the case with GBR bearers) and as such are mapped onto a non-GBR bearer type. However, due to high-priority, an operator may use the ARP parameter to specify the pre-emption capability that allows for the service data flow to be assigned resources that have previously been assigned to another service data flow with a lower priority level. While *real-time messaging services* representing emergency alarms sent to care givers are assigned a priority level of 1 (1*, as noted above), we assign a priority level of 2 to *real-time EHR data access* and *real-time tele-diagnosis* (generally characterized by data and image transfer) in order to distinguish from the priority level assigned to IMS signaling.

On the other hand, we map *non-real-time EHR data access/storage*, *non-real-time tele-diagnosis*, and *non-real-time messaging services *to QCI 6, as such services may be considered more delay tolerant, while being loss-intolerant. While tele-diagnosis services may have high bandwidth requirements due to the transfer of potentially very large medical images, messaging services generally refer to typically low-bandwidth consuming alarms or reminders.

In the case of e-Health services based on research and education, meeting QoS requirements may be considered less critical then in the case of patient care services. With regards to delay requirements and degree of interactivity, we distinguish between the following: *conversational research and education* services mapped to QCI 2 and priority level 4, *streaming research and education* services mapped to QCI 4 (primarily unidirectional data transfer), and *interactive research and education* services mapped to QCI 7 (characterized by a request-response pattern). In the case of services involving the retrieval of health-related information, we distinguish between *interactive health information data exchange* mapped to QCI 8 (e.g., health related web sites involving web browsing), and *non-interactive health information data exchange* mapped to QCI 9 (e.g., background download of health related data). Both QCI 8 and 9 are characterized by loss-intolerance and delay-tolerance, with QCI 8 generally referring to low-priority interactive services and QCI 9 referring to low-priority background services (i.e., are the most delay tolerant). Finally, we identify a class of e-Health services referred to as *administrative and financial transactions* that are mapped to QCI 9 and have generally low bandwidth requirements. Examples include patient referrals, appointment scheduling, charging and billing applications, and transfer of prescription orders.

**Figure 3:**
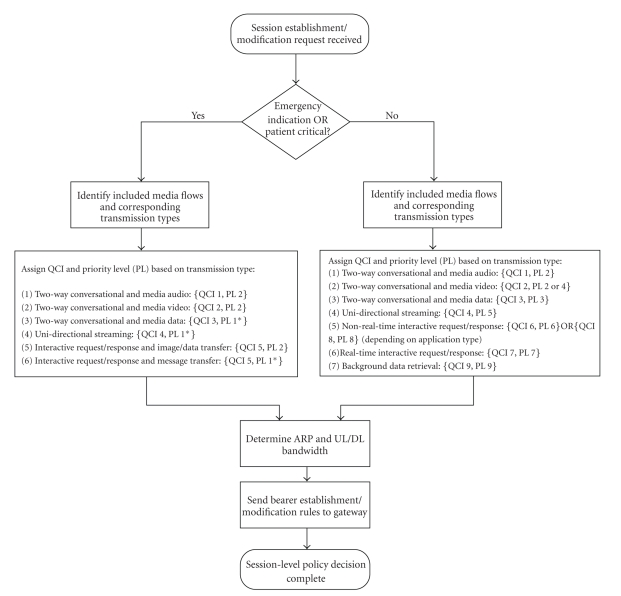
Scheme for assigning QCIs and priority levels to e-Health service flows.

A proposed scheme for assigning QCIs and priority levels to e-Health service data flows is given in Figure 3.

While standards specify performance requirements for each QCI, actual performance that will result if multiple services with a given QCI coexist in the network at the same time will depend on operator dimensioning of network resources for each class, as well as specification of ARP values including pre-emption capability and vulnerability. In that respect e-Health services do not differ from other active services in the network.

## 5. Example

**Figure 4:**
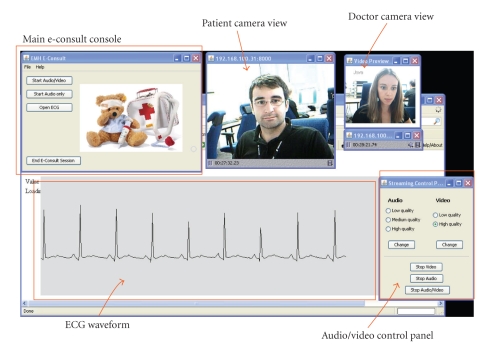
Early research prototype tele-consultation service—doctor desktop view.

In order to illustrate EPS QoS control procedures and the proposed mapping of e-Health service requirements to standardized QCIs, we present a use case involving a tele-consultation service. For the purposes of this paper, the service is referred to as *E-consult* and it involves real-time video conferencing and streaming of ECG signals between a patient and a doctor. The service enables a patient or doctor to initiate an E-consult session using an early research prototype client application (Figure 4 shows the makeshift GUI). The main E-consult console offers the choice of media components to include in the session (audio/video, audio only, and ECG). In case audio/video is selected, two windows with camera views of the “patient” and the “doctor”, respectively, are shown. There is also a user-friendly streaming control panel for selecting audio/video quality and starting and stopping the media flows. The ECG window shows the patient's ECG waveform.

In the prototype application, audio and video streaming corresponds to bidirectional conversational streaming, and it has been implemented using the Java Media Framework (JMF) API [43], which enables the capture, streaming, and transcoding of multiple media formats. We simulate a scenario whereby the patient has access to a remote ECG sensor unit and may choose to transmit ECG signal data to the doctor during the active session. In order to simulate streaming ECG data, we used data available from PhysioBank, a freely available archive of digital recordings of physiological signals to be used for research purposes [44]. The recorded ECG files in PhysioBank used 2-, 3-, and 12-lead ECG records sampled at 500 Hz with 16-bit resolution over a 32 mV amplitude range. For the purpose of our ECG visualization, a small sample of data extracted from ECG recording was stored in a text file in a numerical format.

The network requirements for audio/video correspond to standard requirements for audio and video streaming, with exact values for network parameters depending on the specific type of codec. A streaming control panel included in the E-consult application enables end users to configure preferences with regards to audio and video quality (different chosen quality levels will results in different codecs). Audio quality levels correspond to the following JMF codec settings: (1) low-quality—GSM, (2) medium-quality—ULAW, and (3) high quality—MPEG AUDIO. Video quality levels and JMF settings were specified as follows: (1) low-quality—H.263, and (2) high-quality—motion JPEG.

**Figure 5:**
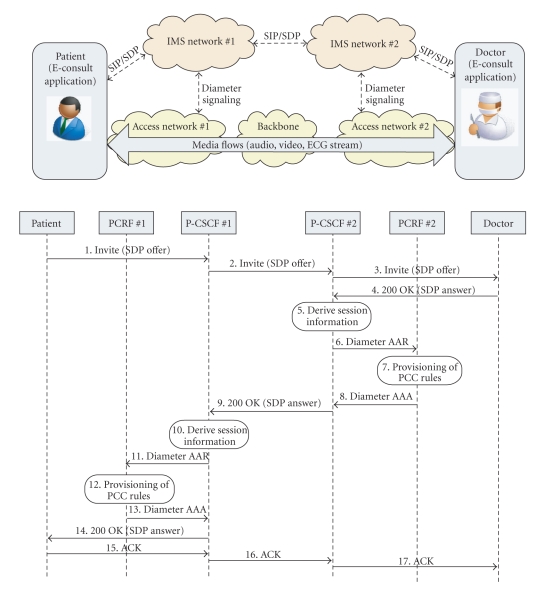
Simplified E-consult network view and session establishment signaling diagram.

A view of the network architecture used for service delivery and a session establishment signaling diagram are given in Figure 5. In the use case involving IMS, both the doctor and the patient would access the E-consult service via their respective access networks and home IMS networks. (We selected this use case since the focus of the paper is on EPS, but in a more general case of end users connecting through their respective access networks through a common core network, QoS agreements among the providers involved in the service delivery chain should exist in order to provide end-to-end QoS.) Service establishment and modification is based on an end-to-end SIP/SDP message exchange via IMS control nodes. Service requirements in terms of media types and bandwidth requirements are specified by the end user application and signaled by using SIP/SDP.

The signaling diagram depicts the patient application as initiating the session by sending a SIP INVITE message including a session description offer specified using SDP. The doctor application replies with a 200 OK message including a subset of supported media types and codecs. In this case, we assume that both end users support specified audio, video, and data formats/codecs. As described in Section 3, the P-CSCF nodes are responsible for extracting session information and invoking authentication and network resource authorization procedures. The PCRF nodes are the functional entities responsible for making session-aware policy decisions and signaling bearer establishment/modification rules to the access network (referred to provisioning of PCC rules in the diagram). This interaction is performed by using an appropriate diameter [45] application protocol (as defined by the 3GPP). Step 7 illustrated in Figure 5 may be executed in parallel with steps 8 and 9, and step 10 in parallel with steps 13 and 14.

In the case of E-consult, the different media flows established as part of the session are mapped to different QCIs due to different QoS requirements. We assume the following mappings: (1) audio stream to QCI 1, (2) video stream to QCI 2, and (3) ECG signal stream to QCI 4. Considering that the example service is not of an emergency nature, there is no need to assign ARP values corresponding to emergency services. On the other hand, if this were to be treated as an emergency situation, then the ECG signal stream would be mapped to QCI 3, and operator policies would determine what ARP values to assign. Since QCI characteristics are specified for the access network (UE to access gateway/PCEF), in the case of two access networks along the end-to-end path, delay values should be summed, and a value for delay in the core network (likely to be much lower) added to it. Based on the findings described earlier, and considering that delay values specified for QCIs represent upper bounds, it may be concluded that the required end-to-end values could be met. Further research and concrete case studies would be needed to validate these conclusions in practice.

## 6. Conclusions and Future Work

Due to a possibly high impact on human life and well-being, e-Health services represent a category of services for which the research on QoS requirements has moved beyond the well-known properties of individual media flows. It has been shown that the context in which the service is invoked may determine the actual classification and prioritization of flows. Our work provides some general guidelines and proposes a mapping of e-Health service types to standardized QCIs in EPS as a next-generation communication technology. A use case of the E-consult service illustrates how the mapping can be applied. Future work will focus on validation of the proposed mapping for selected services.

## Figures and Tables

**Table 1:** QoS requirements for different types of e-Health services with regard to context.

Application type	Required through put	Small delay	Small jitter	Sensitivity to context
Tele-diagnosis	High	Yes	No	Yes
Tele-consultation	High	Yes	Yes	Yes
Tele-monitoring	Low	No	No	Yes
Tele-education	High	No	No	No
Access to EHR	Low/High	No	No	Yes

**Table 2:** Data rates for typical telemedicine devices [7].

Digital device	Temporal/spatial	Contrast/resolution	Required data
	(no. of samples/sec)	(bits per sample)	rate
Digital blood pressure monitor	1	×16	<10 kbps
Digital audio stethoscope	10000	×12	approx. 120 kbps
Electrocardiogram (ECG)	1250	×12	approx. 15 kbps
Ultrasound, cardiology, radiology	512 × 512	×8	256 kB (image size)
Scanned X-ray	1024 × 1250	×12	1.8 MB (image size)
Mammogram	4096 × 4096	×12	24 MB (image size)
Compressed and full motion video	—	—	384 kbps to 1.544 Mbps

**Table 3:** QoS requirements for medical data transfer [8].

Services	Data rate	Maximum delay	Packet loss
Audio	4–25 kbps	150–400 ms	3%
Video	32–384 kbps	150–400 ms	1%
Electrocardiogram (ECG)	1–20 kbps	approx. 1 s	Zero
File transfer (FTP)	Not available	Not available	Zero

**Table 4:** Summary of QoS requirements for e-Health services.

Types of e-Health services	Example e-Health application	Commonly used media types	General QoS requirements	
			Delay	Loss
Real-time conversational tele- consultation	Audio conferencing between patient/doctor or doctor/doctor	Audio	<150 ms E2E one-way	<1% packet loss ratio (PLR) preferred <3% limit

Real-time conversational video- based tele-consultation	Video conferencing between patient/doctor or doctor/doctor	Video	<250 ms E2E one-way (upper bounds reported as 400 ms)	1% PLR

Real-time robotic services	Tele-surgery Tele-ultrasonography	Robotic control data, audio, video	<300 ms round-trip-time	Zero (may tolerate minimal PLR of 0.5%)

Real-time tele- monitoring	Transmission of patient vital signs and streaming video in emergency situations	Biomedical data collected by sensors	Depends on sensors and applications <300 ms E2E one-way for hard real-time ECG (certain applications may tolerate <1 s E2E for ECG)	Zero

Non-real-time tele-monitoring	Transmission of patient vital signs for post- hospital home care	Biomedical data collected by sensors, context data (e.g., collected by environmental sensors)	Not Available (N.A.)	Zero

Real-time tele- diagnosis	Transfer of medical images to remote location in emergency situations	Images, text, data	N.A. (Depends on image size. Smaller images should be transferred within a few seconds.)	Zero

Non-real-time tele- diagnosis	Non-emergency remote diagnosis: transfer of medical images to a remote location where specialists analyze data and return a diagnostic report.	Images, text, data	N.A.	Zero

Real-time EHR data access	Emergency medical personnel at accident/disaster site accessing a patient's EHR	Data, text, graphics, images	N.A.	Zero

Non-real time EHR data access/storage	Web-based end user (patient, doctor, additional health personnel) application for access to EHR during patient check up	Data, text. graphics, images	N.A.	Zero

Real-time messaging	Alarms sent to care givers indicating patient emergency	Text, small images, data	N.A.	Zero

Non-real time messaging	Automated patient alerts (e.g., reminder for check up, reminder to take medication)	Text, small images, data	~10 s [26]	Zero

Conversational research and education	Collaborative research/education tools involving conversational audio/video	Audio, video	<150 ms E2E one-way for audio	<3% PLR audio <1% PLR video
			<250 ms E2E one-way for video (upper bounds reported as 400 ms)	

Interactive research and education	Interactive surgical simulations: remote control of instruments	Data, images	<300 ms round-trip-time	1% PLR

Streaming research and education	Education tools involving streaming media	Audio, video, data	<10 s start up delay for audio and video	<1% PLR audio
				<2% PLR audio

Interactive health information data exchange	Health portals: Web sites offering health related data	All	~2 s/page for Web-browsing [26]	Zero

Non-interactive health information data retrieval	Distribution or diagnostic imaging textbooks	All	N.A.	Zero

Administrative and financial transactions	Patient referrals: appointment scheduling; charging and billing applications	Text	N.A.	Zero

**Table 5:** Standardized QCI characteristics [36].

QCI	Resource type	Priority	Packet delay budget	Packet error loss rate	Example services
1	GBR	2	100 ms	10^−2^	Conversational voice
2		4	150 ms	10^−3^	Conversational video (Live streaming)
3		3	50 ms	10^−3^	Real time gaming
4		5	300 ms	10^−6^	Nonconversational video (buffered streaming)

5	Non-GBR	1	100 ms	10^−6^	IMS signaling
6		6	300 ms	10^−6^	Video (Buffered streaming) TCP-based (e.g., www, e-mail, chat, ftp, p2p file sharing, progressive video, etc.)
7		7	100 ms	10^−3^	Voice, video (Live streaming) interactive gaming
8		8	300 ms	10^−6^	Video (buffered streaming)
9		9			TCP-based (e.g., www, e-mail, chat, ftp, p2p file sharing, progressive video, etc.)

**Table 6:** Rules for derivation of the authorized UMTS QoS parameters from the authorized IP QoS parameters in packet gateway [41].

QCI	Maximum authorized UMTS traffic class and traffic handling priority
1 or 2	Conversational
3 or 4	Streaming
5 or 6	Interactive, maximum authorized traffic handling priority = “1”
	(Signaling indication “yes” for QCI 5; signaling indication “No” for QCI 6)
7	Interactive, maximum authorized traffic handling priority = “2”
8	Interactive, maximum authorized traffic handling priority = “3”
9	Background

**Table 7:** Proposed mapping of e-Health service types to standardized QCIs.

QCI	Priority level	Type of e-Health service	Example e-Health application
1	2	Real-time conversational voice-based tele-consultation	Audio conferencing between patient/doctor or doctor/doctor

2	2	Real-time conversational video-based tele-consultation	Video conferencing between patient/doctor or doctor/doctor
	4	Conversational research and education	Collaborative research/education tools involving conversational audio/video; virtual patient support groups involving conversational audio/video

3	1*	Invasive real-time robotic services	Tele-surgery (transfer of robotic control data in one direction, and streaming images such as ultrasound or video in the other direction)
	3	Non-invasive real-time robotic services	Portable ultrasound probe holder robotic system reproducing an expert's hand movements during an ultrasound examination

4	1*	Emergency real-time tele- monitoring	Tele-monitoring of patient vital signs (e.g., streaming ECG data) in emergency situations
	5	Non-emergency real-time tele-monitoring	Streaming of patient ultrasound images or video during routine check up
		Streaming research and education	Education tools involving streaming media

5	2	Real-time EHR data access	Emergency medical personnel at accident/disaster site accessing a patient's EHR
		Real-time tele-diagnosis	Transfer of medical images to remote location in emergency situations
	1*	Real-time messaging	Alarms sent to care givers indicating patient emergency

6	6	Non-real-time tele-diagnosis	Non-emergency remote diagnosis: transfer of medical images to a remote location where specialists analyze data and return a diagnostic report
		Non-real time EHR data access/storage	Web-based end user (patient, doctor, additional health personnel) application for access to EHR during patient checkup
		Non-real-time messaging	Automated patient alerts (e.g., reminder for check up, reminder to take medication)

7	7	Interactive research and education	Interactive surgical simulations; remote control of instruments

8	8	Non-real-time tele-monitoring	Tele-monitoring application for post-hospital home care of cardiovascular patients involving monitoring of vital signs and delivery to central hospital server
		Interactive health information data exchange	Health portals: Web sites offering health related data

9	9	Noninteractive health information data retrieval	Distribution of diagnostic imaging textbooks
		Administrative and financial transactions	Patient referrals; appointment scheduling; charging and billing applications
